# Safety and Effectiveness of a Novel Liposomal Intra-Articular Lubricant in Symptomatic Knee Osteoarthritis: A First-in-Human Study

**DOI:** 10.3390/jcm13226956

**Published:** 2024-11-18

**Authors:** Shai Shemesh, Oleg Dolkart, Ronit Goldberg, Sabrina Jahn, Amal Khoury, Yaniv Warschawski, Haggai Schermann, Moshe Salai, Gaby Agar, Michael Drexler

**Affiliations:** 1 Assuta Ashdod University Hospital, Ben-Gurion University of the Negev, Ashdod 7747629, Israel; shemesh.shai@gmail.com (S.S.); mt.drexler@gmail.com (M.D.); 2 Liposphere Ltd., Givat-Shmuel 5400804, Israel; ronit.goldberg@lipo-sphere.com (R.G.); sabrina.jahn@lipo-sphere.com (S.J.); 3 Division of Orthopedics, Tel Aviv Sourasky Medical Center, Faculty of Medicine, Tel Aviv University, Tel Aviv 69978, Israel; amalkh@tlvmc.gov.il (A.K.); yanivarsh@gmail.com (Y.W.); 4 Adelson School of Medicine, Ariel University, Ariel 4070000, Israel; sheralmi@gmail.com (H.S.); salaimoshe@gmail.com (M.S.); 5 Sanz Medical Center, Laniado Hospital, Ariel University, Ariel 4070000, Israel; 6 Assuta Hospital Ramat Hachayal, Tel Aviv 6971028, Israel; gabyagar@gmail.com

**Keywords:** knee osteoarthritis, liposomal lubricant, safety, functional outcomes

## Abstract

**Background/Objectives:** Osteoarthritis (OA) is a common disease that affects almost half the population at some point in their lives, causing pain and decreased functional capacity. New conservative treatment modalities are being proposed to provide symptomatic relief and delay surgical intervention. This study aimed at evaluating the safety of the novel liposomal boundary lubricant, injected intra-articularly in patients with moderate knee OA. Additionally, the effect on the functionality and life quality was assessed. **Methods:** Eighteen of the twenty screened subjects met inclusion criteria and were enrolled in the study. After receiving a single IA injection of AqueousJoint, patients were prospectively evaluated at baseline and at 2, 4, 8, 12, and 26 weeks. Numeric Pain Rating Scale (NRS), Knee injury and Osteoarthritis Outcome Score (KOOS), Short Form Health Survey (SF12) and range of motion were also recorded. **Results:** The final analysis was conducted on 18 subjects. No adverse events related to the investigational product were observed in the study. No serious adverse events were observed at all. A significant decrease in pain was demonstrated at all time points vs. baseline (Friedman X^2^ = 35.08, *p* < 0.001). Significant improvement was demonstrated in KOOS pain, symptoms, sports, and ADL subscales (*p* < 0.001). **Conclusions:** Despite a relatively small sample, it was demonstrated that single IA AqueousJoint injection is a safe procedure, resulting in significant pain reduction, higher ADL score, and higher KOOS sport scores. The effects lasted up to 6 months.

## 1. Introduction

Knee OA poses a significant burden on individuals and society, affecting physical, functional, economic, and emotional aspects of daily life [1]. OA is highly prevalent and imposes a substantial economic burden, with costs related to its management and impact estimated to reach up to 2.5% of the gross domestic product [2].

Osteoarthritis (OA) is a prevalent, progressive condition affecting approximately 37% of individuals aged 60 and older, with a higher incidence in women. Characterized by joint pain, stiffness, and reduced mobility, OA leads to significant functional decline and diminished quality of life. Contributing factors include aging, obesity, prior joint injuries, and repetitive occupational stress. These elements exacerbate OA’s impact, underscoring the need for innovative treatments to improve outcomes and reduce its substantial economic and social burden [3].

Even though total knee arthroplasty has been reported to provide successful outcomes in patients with advanced knee OA, [4], patients spend an average of 13.3 years on conservative treatment for symptomatic knee OA before undergoing total knee arthroplasty [5]. Thus, nonsurgical modalities in treating OA have gained an increased interest for younger patients and those without severe degenerative changes [6,7].

Nonsurgical modalities for knee osteoarthritis (OA) are particularly appealing to younger patients due to their desire to maintain an active lifestyle and avoid the risks and longevity concerns associated with surgical interventions. Conservative and minimally invasive treatments such as physical therapy, NSAIDs, and intra-articular injections, including hyaluronic acid and platelet-rich plasma, offer effective symptom relief while delaying or avoiding surgery. These options provide a lower-risk alternative, allowing younger patients to continue their activities with minimal disruption, making nonsurgical approaches an increasingly attractive choice [8,9].

Non-operative treatment options include intra-articular (IA) injections, which have a number of advantages over systemic administration such as higher local bioavailability, reduced systemic exposure, less adverse events (AEs), and lower costs [10,11]. Traditional injections with corticosteroids and hyaluronic acid (HA) are largely used in the clinical practice, although conclusions regarding their clinical utility are inconsistent, repeated injections are needed, long-term effects remain unclear, and they may be associated with AEs [11,12].

Surface-active boundary lubricants that coat the articular surface of the joint might be a promising strategy for the treatment of OA and a potential alternative to IA-HA injections. Liposomes have been commonly studied and commercialized in the context of drug delivery, as well as for IA applications [13]. The mode of action of these liposomal boundary lubricants is based on the hydration lubrication mechanism [14].

AqueousJoint is a new IA injectable joint lubricant for patients suffering from knee OA developed by Liposphere LTD. AqueousJoint is a liposomal boundary lubricant, which coats the cartilage surface and protects it from further damage and degradation [15]. AqueousJoint is a surface lubricant that does not increase the synovial fluid viscosity but provides mechanical protection from cartilage degradation and wear [15]. IA AqueousJoint injection demonstrated an excellent safety profile and did not result in local reactivity or systemic toxicity [16].

This first-in-human study was designed to evaluate the safety of IA injection of AqueousJoint in OA patients during up to 6 months follow-up. Additionally, the effect of the treatment on functionality, range of motion, life quality, and analgesic consumption was assessed.

## 2. Methods

### 2.1. Study Design

This study was designed as a multicentre (two centres), open-label, safety clinical trial. The study was conducted from June 2022 to January 2023. The study protocol, its amendments, and the patient informed consents were reviewed and approved by the appropriate independent Ethics Committees. This study was registered at clinicaltrials.gov (NCT05412836).

A formal sample size calculation was not performed for this first-in-human, safety-focused trial. The sample size was intentionally kept small to enable close monitoring of participants and ensure detailed safety assessments. This approach aligns with the exploratory nature of initial safety studies.

### 2.2. Study Population

Eligible participants, 40–80 years old men and women diagnosed with primary knee OA who met the inclusion criteria, were enrolled. Major inclusion criteria were: pain in the intended study knee with an average NRS score of >5 over the last week prior to visit 1 (pre-injection); degenerative changes in the intended study knee that can be categorized as grade II-IV Kellgren–Lawrence based upon standing posterior–anterior and lateral radiographs of the knee based on X-ray from up to the last 6 months prior to the first visit (pre-injection); and Body Mass Index (BMI) between 18.5 and 35. The exclusion criteria were: history of significant knee trauma or previous arthroscopic surgery of the intended study knee within the last 3 months preceding screening; concomitant moderate or large size synovial fluid effusion of the index knee at screening; fever signs or symptoms of systemic infection or infection of the intended study knee, on the day before or the day of administration of treatment; IA injection to the intended study knee within 3 months prior to screening; intake of chronic pain medications (especially opioid pain relievers) without an option to pause for the period of the study.

Patients eligible at screening entered a washout period for analgesics and NSAIDs lasting 2 to 5 days, depending on the drug. Patients were prohibited to use regular analgesics, glucosamine or chondroitin, or NSAIDs during the study. Paracetamol 500 mg; maximum daily dose 4 g, was the only rescue medication allowed for use during the study. Paracetamol was not permitted to use during the 24 h period prior to each study visit.

### 2.3. Interventions

After signing the informed consent, patients received a single IA injection of AqueousJoint (4 mL) into the target knee. The choice of the injection volume was based on the amount of standard sodium hyaluronate products that are commonly injected several times at weekly intervals. All procedures were performed in an outpatient clinic by two senior authors, who have experience of >100 cases per year in knee joint injections or aspirations. Patients were placed in a supine position. The knee was flexed approximately 60° and prepared in a sterile fashion. A 21-gauge needle (0.8 × 50 mm) was inserted into the joint capsule.

### 2.4. Patients’ Evaluation

All patients were clinically evaluated before the AqueousJoint injection and at the follow-up visits at 2, 4, 8, 12, and 26 weeks. All complications and adverse events were documented during patient assessment. The primary clinical outcome was to evaluate the safety of IA injection of AqueousJoint. The safety parameters included: adverse reactions related to the injected material; and injection-related side effects consisting of injection-site reaction, erythema, swelling, injection-site pain, and pruritus.

To assess the effectiveness of AqueousJoint injection, further scores were used, including the Numeric Pain Rating Scale (NRS), Knee injury and Osteoarthritis Outcome Score (KOOS), and Short Form Health Survey (SF12).

A further analysis of the clinical effectiveness of the treatments was performed, assessing the number of patients that reached the minimal clinically important difference (MCID) for the NRS and KOOS subjective scores.

All patients were followed up for 26 weeks. The study consisted of the following visits: screening (0); a baseline (1) for injecting IA AqueousJoint in the study knee; 3 phone-call visits that were conducted at day 2 and 4 and 3 weeks post-injection; six follow-up visits that took place at 1, 2, 4, 8, 12, and 26 weeks post-injection.

To ensure participant compliance, a structured follow-up schedule was implemented, supplemented by phone check-ins at key time points. Participants maintained study diaries to log daily medication use and symptoms, which were reviewed during follow-up visits. Compliance with protocol restrictions on analgesics and NSAIDs was reinforced at each visit, with only paracetamol allowed as rescue medication. Prior to the study, participants received detailed instructions to ensure understanding and adherence to the protocol.

### 2.5. Statistical Analysis

Data were analysed using SPSS version 27. Descriptive statistics were produced using frequencies (N/%) for categorical variables (e.g., sex) and means with standard deviations for continuous variables (e.g., age). To analyse the effectiveness measures of the treatment, the per-protocol approach was used. The per-protocol approach includes only participants who received the treatment as intended, without any significant deviations or protocol violations, and provided data for all study visits. To increase the reliability of the results, data were analysed using a per-protocol method, a within-subject design. In this study, statistical analyses were conducted to evaluate the primary and secondary outcomes, ensuring that results were accurately interpreted and significant findings were reliable. The Friedman test was used as the primary analysis tool for assessing differences in repeated measures across multiple time points. This non-parametric test was selected due to its robustness in handling data that do not meet the assumptions of normality, which is often a concern in small sample sizes [17].

The effectiveness of the treatment was analysed for every outcome separately in two stages. First, the trend of change was analysed by visits using the Friedman test, which detects differences in outcomes across multiple measurements conducted on the same subjects. Second, in case of detection of a significant trend, Wilcoxon procedures were performed, testing differences between the score at the baseline and the score at every follow-up visit. The basic *p*-value was 5%. The *p*-value of the Friedman test was adjusted to multiple comparisons.

## 3. Results

Among the 20 screened subjects, 18 patients met the inclusion criteria and were included in the study, predominantly males (68.4%). The average age was 68.79 (SD = 7.75), and the average BMI was 29.75 (SD = 3.67). Baseline characteristics of the study cohort are described in Table 1.

**Figure 1:**
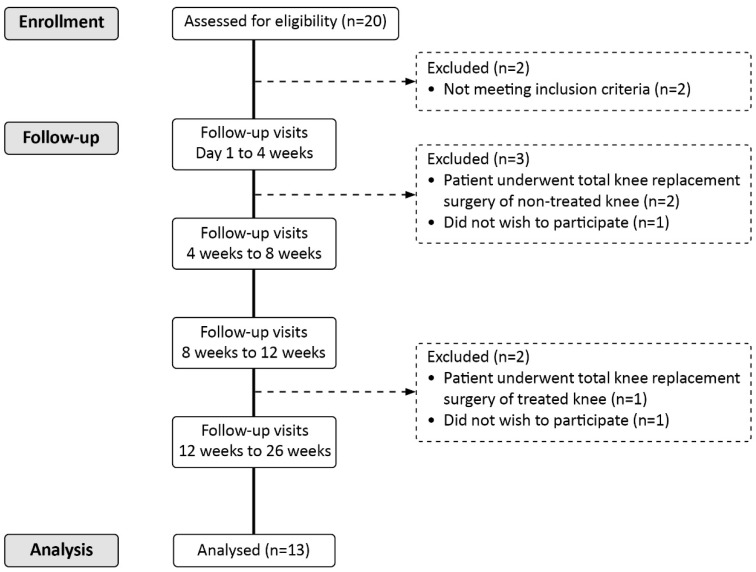
CONSORT.

The CONSORT (Consolidated Standards of Reporting Trials) flow diagram showing patients’ inclusion and follow-up is reported in Figure 1.

## 4. Safety

There were no adverse effects, nor serious adverse effects related to the product. No adverse reactions related to the injected product such as injection-site reactions including erythema, swelling, injection-site pain, or pruritus were reported. A total of 12 mild adverse events were observed. They included muscle pain after physical activity, toe numbness, etc. All these adverse events were treatment unrelated, self-limiting, and none required any specific procedure or hospitalization.

### 4.1. Effectiveness

#### NRS

**Figure 2:**
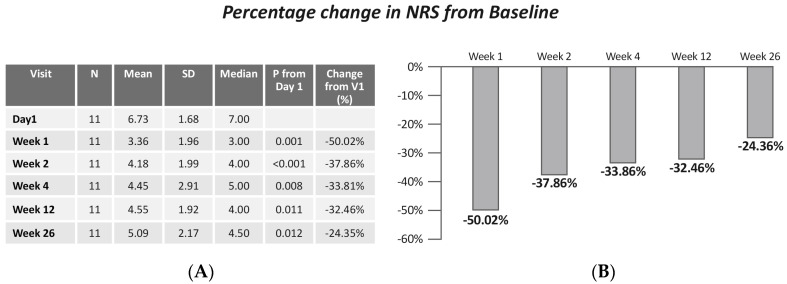
(**A**) NRS scores by visits; (**B**) change in NRS from baseline. Values are presented as mean ± SD.

A significant decrease in NRS pain score was demonstrated (Friedman X^2^ = 35.08, *p* < 0.001). Specifically, results showed a significant reduction between NRS at baseline and NRS at day 4 (*p* = 0.002), week 1 (*p* = 0.001), week 2 (*p* < 0.001), week 3 (*p* = 0.005), week 4 (*p* = 0.008), week 8 (*p* = 0.003), week 12 (*p* = 0.011), and week 26 (*p* = 0.012) (Figure 2).

### 4.2. KOOS

**Figure 3:**
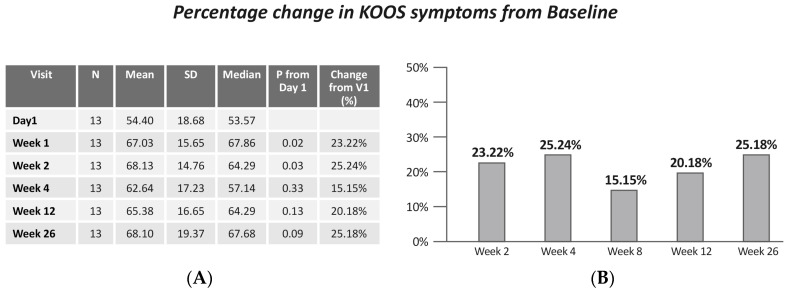
(**A**) KOOS symptoms by visits; (**B**) change in KOOS symptoms from baseline.

A significant improvement in KOOS Symptoms was demonstrated (Friedman X^2^ = 9.89, *p* = 0.042). Specifically, a significant improvement was shown between baseline and week 2 (*p* = 0.02) and week 4 (*p* = 0.03) (Figure 3).

**Figure 4:**
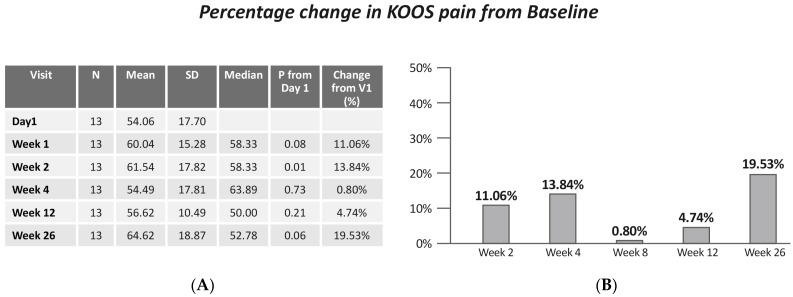
(**A**) KOOS pain by visits; (**B**) change in KOOS pain from baseline. Values are presented as mean ± SD.

No statistically significant improvement in KOOS pain was demonstrated (Friedman X^2^ = 9.90, *p* = 0.078). However, a significant improvement in pain was detected between baseline and week 4 (*p* = 0.01) (Figure 4).

**Figure 5:**
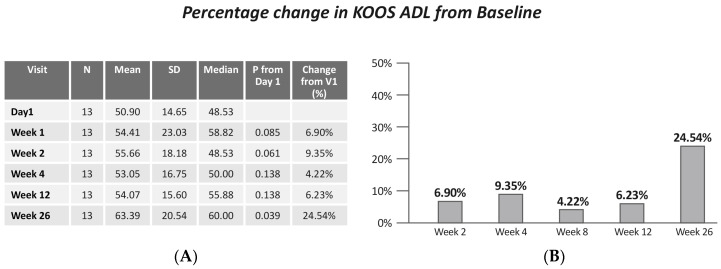
(**A**) KOOS ADL by visits; (**B**) change in KOOS ADL from baseline. Values are presented as mean ± SD.

A significant improvement in KOOS ADL (Friedman X^2^ = 12.95, *p* = 0.024) was revealed. Specifically, the improvement was demonstrated increase between baseline and KOOS ADL at week 26 (*p* = 0.039) (Figure 5).

**Figure 6:**
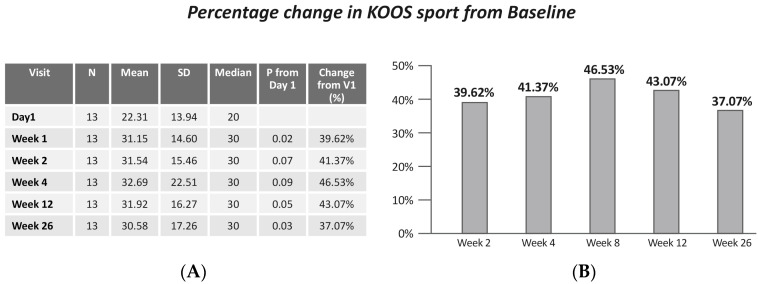
(**A**) KOOS sport by visits; (**B**) change in KOOS sport from baseline. Values are presented as mean ± SD.

A significant improvement in KOOS sports activity was demonstrated between day 1 and week 2 (*p* = 0.02), week 12 (*p* = 0.05), and week 26 (*p* = 0.03) (Figure 6).

**Figure 7:**
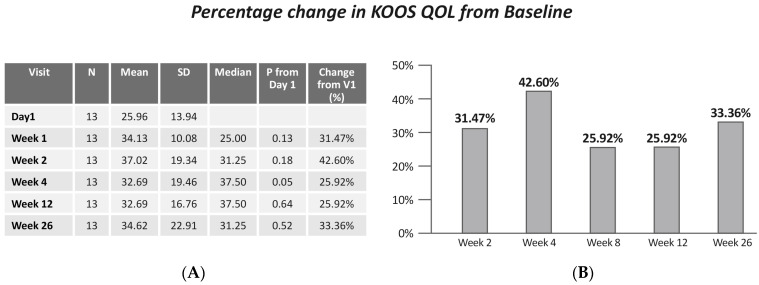
(**A**) KOOS QOL by visits; (**B**) change in KOOS QOL from baseline. Values are presented as mean ± SD.

No statistically significant change was found in KOOS QOL (Friedman X^2^ = 4.01, *p* = 0.40). However, a significant improvement was found between KOOS QOL at baseline and QOL at week 8 (*p* = 0.05) (Figure 7).

#### SF 12

No statistically significant change was found in SF 12 during the study period in both physical and mental sub scores ((Friedman X^2^ = 6.30, *p* = 0.278) and (Friedman X^2^ = 2.51, *p* = 0.775), respectively).

## 5. Discussion

The principal results of this open-label safety trial, conducted at two centres, revealed that single IA injection of AqueousJoint, administered to patients with moderate knee OA, is a safe procedure. No adverse events, nor serious adverse events, related to the investigational product were reported. In addition, this study showed that a single injection of AqueousJoint provided statistically significant relief of pain and symptoms that lasted for up to and including 6 months.

Although lipid-based formulations are being used and developed for IA injections to treat OA, very little information is available on their local and systemic toxicity in vivo and in clinical trials. There is a single study that examined preclinical toxicity of pMPCylated liposomes injected IA in rats and sheep knees [16]. They concluded that a single IA injection of pMPCylated liposomes is safe and does not result in local reactivity or systemic toxicity in rats and in sheep [16].

Indeed, this trial was the first one that investigated the effects of an IA AqueousJoint injection in patients with knee OA. Safety was the major point of interest in the current study. The reported adverse event was muscle pain after enhanced physical activity. It can be assumed that muscle pain is most probably related to the vigorous workout, and not related to the local IA injection. There were a few single episodes of weakness, hypotension, pneumonia, and toe numbness, all of which were not related to the investigational product. Two patients underwent total knee arthroplasty of the contralateral knee. No adverse reactions related to the injected product such as injection-site reactions including erythema, swelling, injection-site pain, or pruritus were reported. Overall, AqueousJoint demonstrated an excellent tolerability when injected IA in knee joint.

In the current study, the use of AqueousJoint, a novel liposomal boundary lubricant, has been associated with clinical effectiveness (in terms of pain relief) for patients with moderate symptomatic knee OA. This benefit was achieved at early time points (one week after the injection) and maintained for at least 26 weeks. In general, clinical improvement has been established with a minimum difference of 20% in effectiveness outcomes [18], like scores in the KOOS questionnaire.

The mode of action of AqueousJoint involves four key mechanisms: (a) Liposome Hydration: Each liposome is highly hydrated in physiological solution due to the formation of tightly bound hydration shells around the phosphocholine groups of the lipid-based structure, creating a lubricating water layer around the particle [14]. (b) Liposomal Boundary Layer Formation: AqueousJoint forms a stable, multi-layered liposomal boundary on the cartilage surface, reducing friction and protecting against mechanical wear via the Hydration Lubrication Mechanism. (c) Stability and lubrication under High Loads: Phosphatidylcholine-based liposomes, due to their structural arrangement on the surface, maintain superior lubrication even under high loads and shear, acting as molecular “ball bearings” [19]. (d) Hydration Lubrication Mechanism (HLM): The combination of these above-mentioned factors allows for effective load-bearing and low-friction sliding, resulting in reduced joint wear and improved mobility. These unique properties provide direct mechanical cartilage protection, offering a distinct advantage over traditional injectables like hyaluronic acid, which primarily work by modifying synovial fluid viscosity [20].

In contrast to intra-articular HA and corticosteroids, AqueousJoint is a liposomal boundary lubricant which directly coats the cartilage surface and protects it from further damage and degradation during articulation and, subsequently, alleviates pain in OA patients. The mechanism of action of AqueousJoint is mechanical in nature through an entropy-favoured process [21].

The current study effectiveness results are comparable with the recently published results by several RCTs reporting clinical results in patients with knee OA treated with IA injections. A recent study by Jalal Jivan S et al. compared the effectiveness of IA injection of platelet-rich plasma (PRP) with HA (as one of the standard treatments) on mild to moderate knee OA [22]. They found that in the majority of visits (especially 6 and 12 month), the extent of improvement in scores of KOOS subscales was significantly higher in the PRP recipients compared to that in the HA recipients. The change in KOOS pain in the PRP group at 6 month was 15.6 vs. 19.53 in the current study. Another study by Filardo G et al. examined the benefit provided by PRP injections to treat knee joint degeneration in comparison with HA [23]. The current study demonstrated higher improvement in all KOOS subscales as compared to both PRP and HA groups in the study by Filardo G et al. [23].

## 6. Limitations

Since it was a first-in-human safety study, this study did not include a control or comparator group. Although the treatment of OA has an important placebo effect [24], and recognising that a control group would have strengthened the conclusions, results are in concordance with controlled studies, revealing a superior impact of HA injections. Another limitation was related to the limited sample size of the study (n = 18). The follow-up period was relatively short as the most significant change was expected to occur within the first 6 month following injection. Patients were prohibited from using regular analgesics and NSAIDs during the trial period to minimize confounding effects, which may limit the generalizability of the findings to those who typically rely on these medications for daily pain management in clinical practice.

Additionally, while the 26-week follow-up period provided medium-term insights, longer-term follow-up is necessary to assess the durability of treatment effects and long-term safety. Simplifications in this study included the exclusion of biomarkers and advanced imaging techniques, limiting the ability to objectively evaluate cartilage integrity. Only a per-protocol analysis was conducted, which may not reflect real-world adherence. To address these limitations, future research will include larger, randomized controlled trials with extended follow-up periods, the incorporation of biomarkers and imaging studies, and the use of intention-to-treat analyses to enhance the robustness and applicability of the results.

## 7. Conclusions

Based on the data collected in this safety study, it can be concluded that a single intra-articular injection of 4 mL of AqueousJoint at a concentration of 10 mM, administered to patients with knee osteoarthritis, is a safe procedure. No adverse or serious adverse events related to the investigational product were observed in the study.

In addition, this study showed that a single injection of AqueousJoint provided statistically significant relief of pain and symptoms for up to and including 6 months.

## Figures and Tables

**Table 1:** Baseline demographic characteristics of the subjects.

Baseline Demographic Parameters, *n* = 18			
Parameter		Mean	SD
Kellgren-Lawrence grade	II	1	NA
	III	17	NA
Gender	Female	6	NA
	Male	12	NA
Age		68.79	7.75
Weight		83.68	12.35
Height		167.63	9.78
BMI		29.75	3.67
VAS		6.3	2.08

## Data Availability

The datasets used and/or analysed during the current study are available from the corresponding author on reasonable request.
